# Benign enlargement of the subarachnoid spaces and subdural collections—when to evaluate for abuse

**DOI:** 10.1007/s00247-023-05611-y

**Published:** 2023-03-01

**Authors:** Maria Raissaki, Catherine Adamsbaum, Maria I. Argyropoulou, Arabinda K. Choudhary, Annmarie Jeanes, Kshitij Mankad, Inès Mannes, Rick R. Van Rijn, Amaka C. Offiah

**Affiliations:** 1 Department of Radiology, University Hospital of Heraklion, Medical School, University of Crete, Crete, Greece; 2 Emeritus Pediatric Radiologist, Faculty of Medicine, Paris-Saclay University, 63 Rue Gabriel Péri, 94270 Le Kremlin Bicêtre, France; 3 Department of Clinical Radiology and Imaging, Medical School, University Hospital of Ioannina, Ioannina, Greece; 4 Department of Diagnostic Radiology, University of Arkansas for Medical Sciences, Little Rock, AR USA; 5 Department of Paediatric Radiology, Leeds Children’s Hospital, Leeds Teaching Hospitals NHS Trust, Leeds, UK; 6 Department of Radiology, Great Ormond Street Hospital, London, WC1N 3JH UK; 7 Pediatric Radiology Department, AP-HP, Bicêtre Hospital, Le Kremlin‑Bicêtre, France; 8 Department of Radiology and Nuclear Medicine, Emma Children’s Hospital, University of Amsterdam, Amsterdam UMC, Amsterdam, the Netherlands; 9 Department of Oncology & Metabolism, University of Sheffield, Sheffield, UK

**Keywords:** Abusive head trauma, Benign enlargement of the subarachnoid spaces, Infants, Macrocephaly, Magnetic resonance imaging, Subdural collections, Ultrasound

## Abstract

In infants without a history of trauma, subdural haemorrhages should raise the concern for an abusive head injury, particularly when they are associated with bridging vein clotting/rupture or with septations. However, non-haemorrhagic, fluid-appearing subdural collections (also called hygromas) may also be the result of abuse. Subdural collections have also been uncommonly observed in patients with benign enlargement of the subarachnoid spaces (BESS) and a few large-scale studies accurately investigate the incidence and the significance. Currently, there is a wide variation of practices in children with BESS and subdural collections. Due to the social risks associated with abuse evaluation and the perceived risk of radiation exposure, there might be a reluctance to fully evaluate these children in some centres. The diagnosis of physical abuse cannot be substantiated nor safely excluded in infants with BESS and subdural collection(s), without investigation for concomitant traumatic findings. The exact prevalence of occult injuries and abuse in these infants is unknown. In macrocephalic infants with subdural collections and imaging features of BESS, thorough investigations for abuse are warranted and paediatricians should consider performing full skeletal surveys even when fundoscopy, social work consult, and detailed clinical evaluation are unremarkable.

## BESS: Nomenclature, typical clinical, and imaging findings

Benign enlargement of the subarachnoid spaces (BESS) is one of the causes of macrocephaly in infants and a self-limiting condition in most cases. It can be defined as an increased or increasing head circumference, with a widened subarachnoid space on neuroimaging and no other cause accounting for macrocephaly. In infants with a normal, small or gradually reducing head circumference percentile and widened subarachnoid spaces, the diagnosis of BESS should not be considered and other causes of brain underdevelopment or even atrophy secondary to numerous aetiologies might be considered.

BESS most commonly affects boys and the incidence is 0.4 per 1,000 live births (95% confidence interval, 0.34 to 0.46) [1, 2]. Infants with BESS present with macrocephaly (head circumference more than two standard deviations above the mean compared to international standards [3]), often above the 90^th^–98^th^ percentile, at the age of 3–12 months peaking around 7 months. These children may have a family history of macrocephaly, they are born normocephalic or macrocephalic and a vast majority of them are developmentally normal both at presentation and at follow-up [4–6]. The enlarged subarachnoid spaces subside by 1–2 years of age while macrocephaly stabilises and persists along a curve parallel to the 95–98% curve [7]. A small percentage may show transient developmental delay [2] while a non-negligible percentage may end up with motor and verbal delays at pre-school age [8].

Benign enlargement of the subarachnoid spaces (BESS) has also been called benign familial hydrocephalus, benign external hydrocephalus, benign enlargement of the extra-axial spaces, idiopathic external hydrocephalus, benign extra-axial/extracerebral collections of infancy, extra-ventricular hydrocephalus, pseudo-hydrocephalus, benign communicating hydrocephalus, and extra-ventricular obstructive hydrocephalus [9, 10]. Subdural hygroma, benign subdural effusion, benign hygroma of infancy, although they are currently used for collections in a different space, the subdural space, are sometimes inappropriately used to describe BESS, adding to the confusion [11].

Hypotheses regarding the pathogenesis of BESS include a delayed maturation of arachnoid villi leading to defective absorption of cerebrospinal fluid (CSF) and consequent CSF accumulation in the subarachnoid spaces, and/or amplification of the physiologic imbalance between the skull and brain growth in infants between 3 months and 1 year of age [12]. Subarachnomegaly-venous congestion of infancy has recently emerged as a term to indicate the correct location of prominent CSF space and implicates venous outflow impairment as a possible pathogenetic mechanism [7]. Magnetic resonance (MR) venogram may show hypoplasia of transverse sinuses in subarachnomegalic patients [7, 13]. In infants, arachnoid granulations are not completely developed and the intradural vascular plexus is larger; these, together with meningeal lymphatic vessels in the dura matter appear to play a role in CSF absorption, although further studies are required to demonstrate age-related changes of CSF outflow in humans [14].

**Fig. 1:**
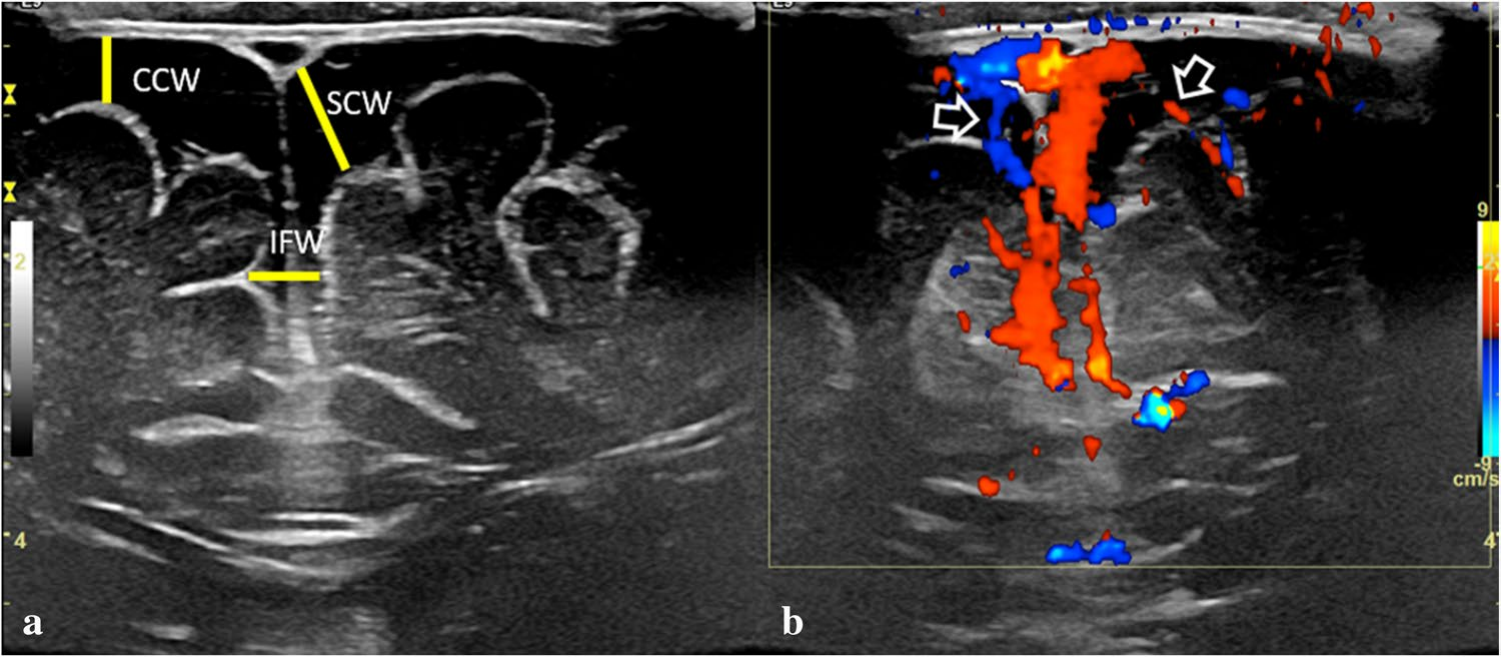
Brain ultrasound, coronal scans of a 6-month macrocephalic boy with normal psychomotor development and a family history of macrocania (both parents), diagnosed as benign enlargement of the subarachnoid spaces. **a** Coronal image with a 15 MHz linear probe through the anterior fontanelle shows increased cranio-cortical width (the widest vertical distance between brain surface and calvarium), increased sino-cortical width (the widest distance between lateral wall of superior sagittal sinus and cortical surface) and moderately wide interhemispheric fissure width (the widest horizontal distance between hemispheres), all ≥ 5 mm. **b** Colour Doppler, shows multiple vessels (*arrows*) traversing the subarachnoid space CCW: Cranio-cortical width SCW: Sino-cortical width IFW: interhemispheric fissure width

**Fig. 2:**
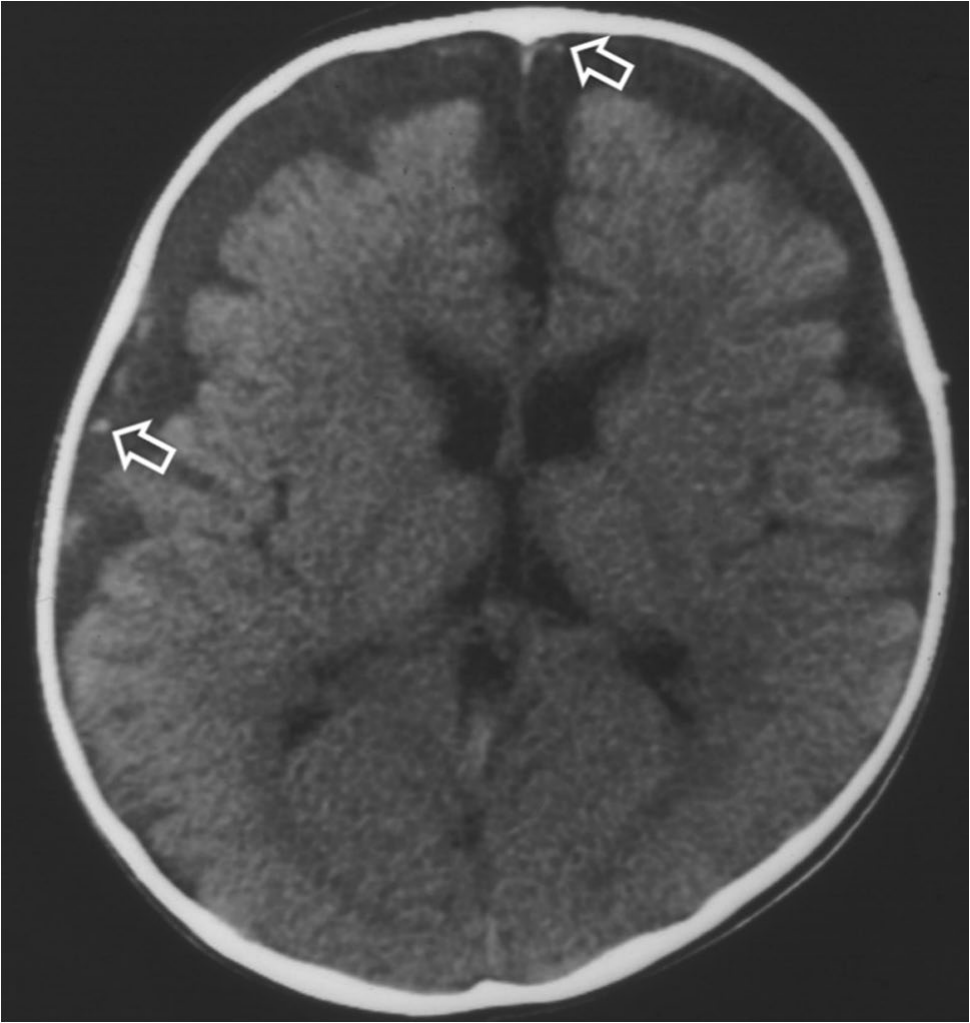
Axial brain computed tomography image of a 7-month-old boy with macrocrania and benign enlargement of the subarachnoid spaces. There is an enlargement of the subarachnoid spaces over the frontal lobes and at the interhemispheric fissure without cortical compression. Importantly, the vessels are located away from the cortical surface of the brain (*arrows*)

**Fig. 3:**
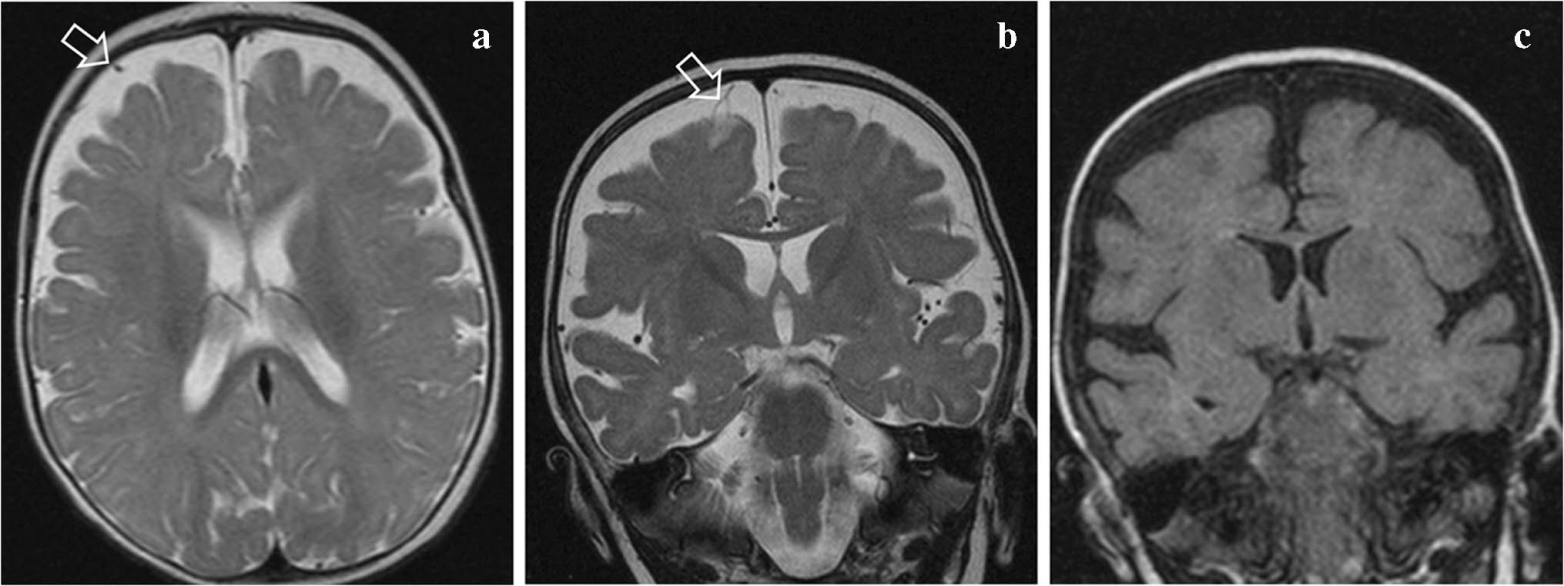
Brain magnetic resonance imaging of a 7-month-old male with a large head and seizures lasting less than 5 min. **a** Axial and (**b**) coronal T2-weighted scans show prominence of Cerebrospinal fluid (CSF) spaces over the frontal lobes with vessels traversing the entire width of the subarachnoid space (*arrows*). **c** Coronal fluid attenuation inversion recovery image shows extracerebral fluid with isointense signal compared to the CSF

Typical imaging appearances in BESS include excess CSF in the subarachnoid space, overlying both frontal lobes and extending into a widened anterior interhemispheric fissure. Occasionally, widened Sylvian fissures and a normal or only slight increase in the volume of the lateral ventricles may be seen [2, 8, 10, 15, 16]. At present, there are no imaging criteria for BESS and no established cut-off values [17]. Age-dependent sinocortical, craniocortical, and interhemispheric widths above the 95^th^ percentile are considered abnormal [18, 19]. Upper limits of normal craniocortical width have reportedly ranged from 4 to 10 mm in infants (<1 year of age) and 3.3 to 5 mm in neonates [17]. Craniocortical width of  >5 mm is considered widened and  >10 mm significantly widened, regardless of modality used, ultrasound (US), computed tomography (CT), or MR imaging (MRI) [15, 18–21]. In BESS, in contrast to subdural hygromas, fluid does not cause a mass effect upon the brain, vessels are elongated and cross the entire width of the enlarged subarachnoid space, without being displaced towards the gyri [21, 22]. On US, there should be no cortical flattening, no midline shift, no areas of increased echogenicity within the CSF and no visible arachnoid membrane (Fig. 1). In children with BESS, on CT and MRI images, vessels in the subarachnoid spaces appear thin, non-displaced towards the gyri, without evidence of adjacent clots (Figs. 2 and 3).

## Multimodality differentiation between enlarged subarachnoid spaces and subdural collections

Subdural collections can appear unicompartmental without septations exhibiting a homogeneous or heterogeneous echogenicity, density, or intensity, while multicompartmental subdural collections with septations and heterogeneous components may also occur. Different terms including haematoma, haematohygroma, chronic haematoma, and hygroma have been used in the literature to describe these imaging patterns in a subdural collection. These imaging appearances are attributed to the presence of clotted and unclotted blood, mixture of blood with CSF following bridging vein injury and arachnoid tear, and the presence of neomembranes [21].

**Fig. 4:**
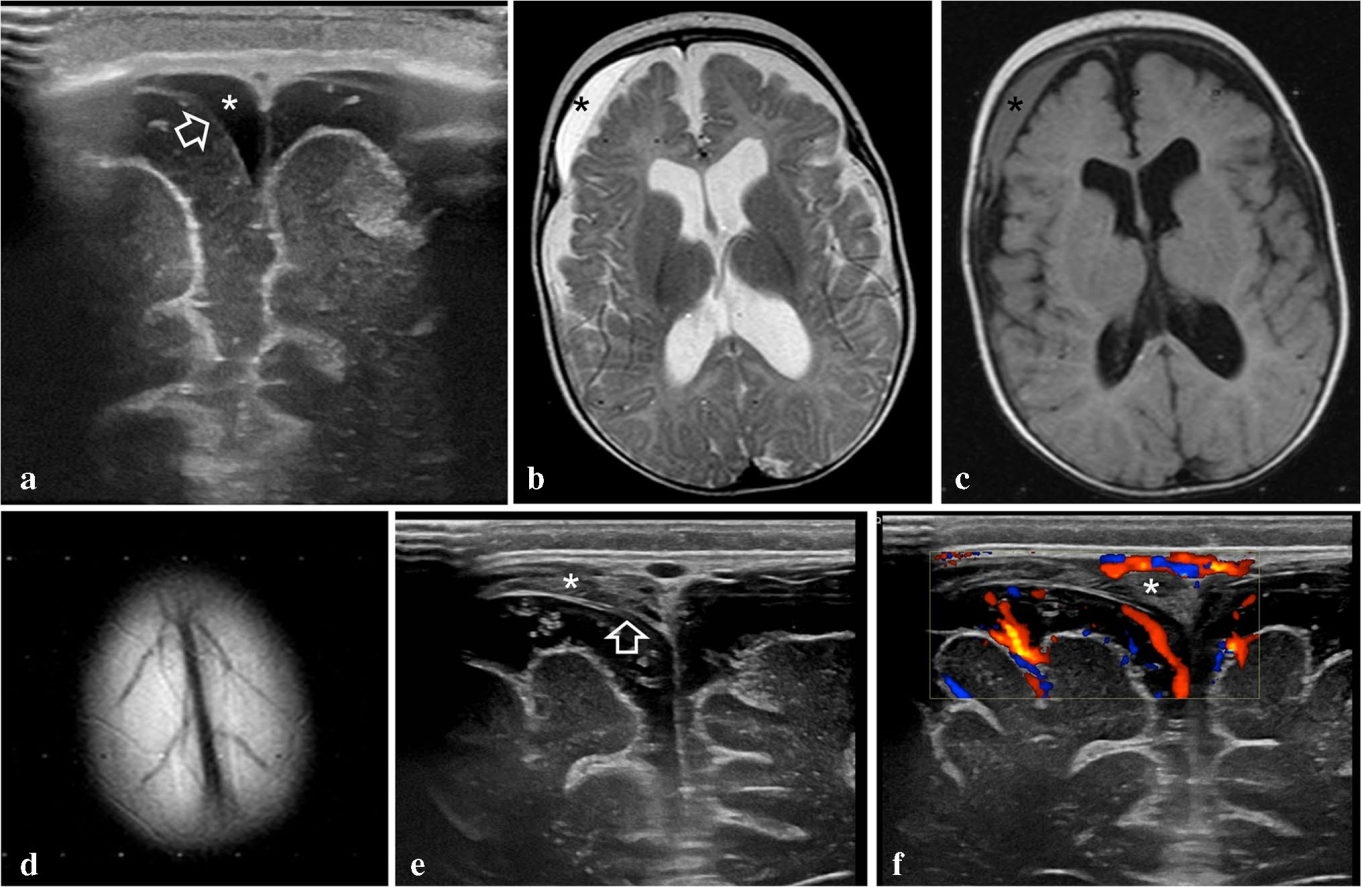
A 3-month-old ex-premature boy with progressive macrocrania and normal development. On ultrasound (US), there was an incidentally discovered subdural collection, confirmed by magnetic resonance imaging (MRI). Fundoscopy, skeletal surveys, social and family history, and clinical follow-up were unremarkable. The diagnosis was subdural collection in the setting of benign enlargement of the subarachnoid spaces. **a** Coronal US with a high-frequency linear transducer shows enlargement of subarachnoid spaces bilaterally and an anechoic subdural collection (*) displacing the arachnoid dura (*arrow*) on the right. A small linear membrane was also seen on the left. **b** MRI 2 weeks later. Axial T2-weighted image confirms increased craniocortical width and the presence of a hyperintense subdural collection (*). **c** MRI, axial FLAIR image, same level as in b. The subdural collection (*) is more conspicuous and restricts the adjacent subarachnoid space. **d** Axial T2-weighted image at thew convexity shows normal bridging veins. **e** Repeat ultrasound scan on the same day shows increased echogenicity of the subdural collection (*) while the arachnoid membrane is still visible (*arrow*). **f** Coronal colour doppler US of the same area confirms lack of vessels within the collection (*)

**Fig. 5:**
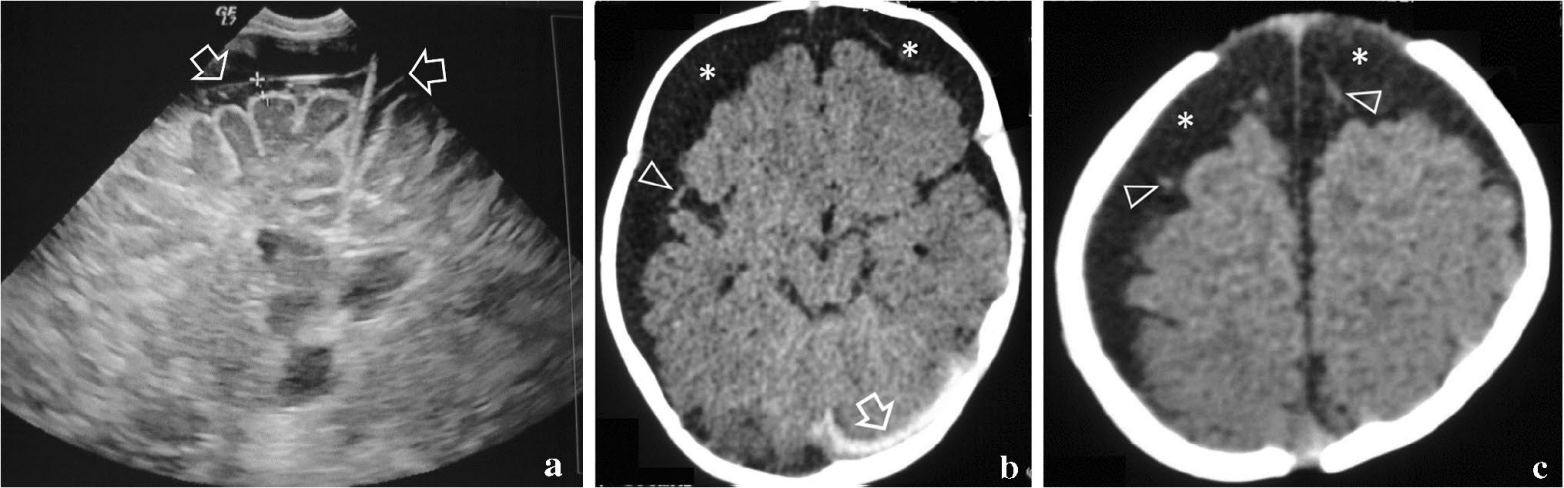
A 3.5-month-old abused boy was shaken and thrown against the floor in public by his alcoholic father. He was brought to hospital by the police with irritability, unalert gaze, bulging anterior fontanelle, generalised hypertonia, and a head circumference above the 97th percentile. **a** Coronal ultrasound, showing visible arachnoid membranes bilaterally (*arrows*), clearly separating the pericerebral spaces into subdural and subarachnoid (*between cursors*) compartments. **b, c** Axial computed tomography scan images demonstrate convexity subdural collections with cerebrospinal fluid density (*), a hyperdense infratentorial subdural haematoma (*arrow*) and subarachnoid vessels displaced against the brain’s surface (*arrowheads*). Such imaging findings should always rise concerns for trauma and should not be misinterpreted for benign enlargement of subarachnoid spaces

On US, it is important to closely inspect the convexity with linear probes, colour Doppler and/or power Doppler or B-flow techniques. In BESS, a widened subarachnoid space containing crossing bridging veins is seen on colour Doppler (Fig. 1). In subdural collections, an excess line parallel to the dura, represents the arachnoid membrane which is normally not visible (Figs. 4 and 5). Moreover, the subarachnoid vessels are displaced towards or against the cortical surface, while differences in fluid echogenicity and space-occupying phenomena on the brain surface may exist (Figs. 4 and 5) [22].

On CT, subdural haematomas can be clearly identified when hyperdense and less conspicuous when isodense is related to grey matter. It can be difficult to differentiate between BESS and subdural hygromas because there might be no difference in density between CSF in BESS, almost pure CSF in hygromas (Fig. 5) and CSF mixed with few blood products in haematohygromas following rupture of the arachnoid membrane and mixture of CSF with blood [23–25]. It can be useful to use a subdural (blood) window which may increase the sensitivity to detect thin subdural haematomas (centre/level 50–100 HU; width 130–300 HU) [26].

**Fig. 6:**
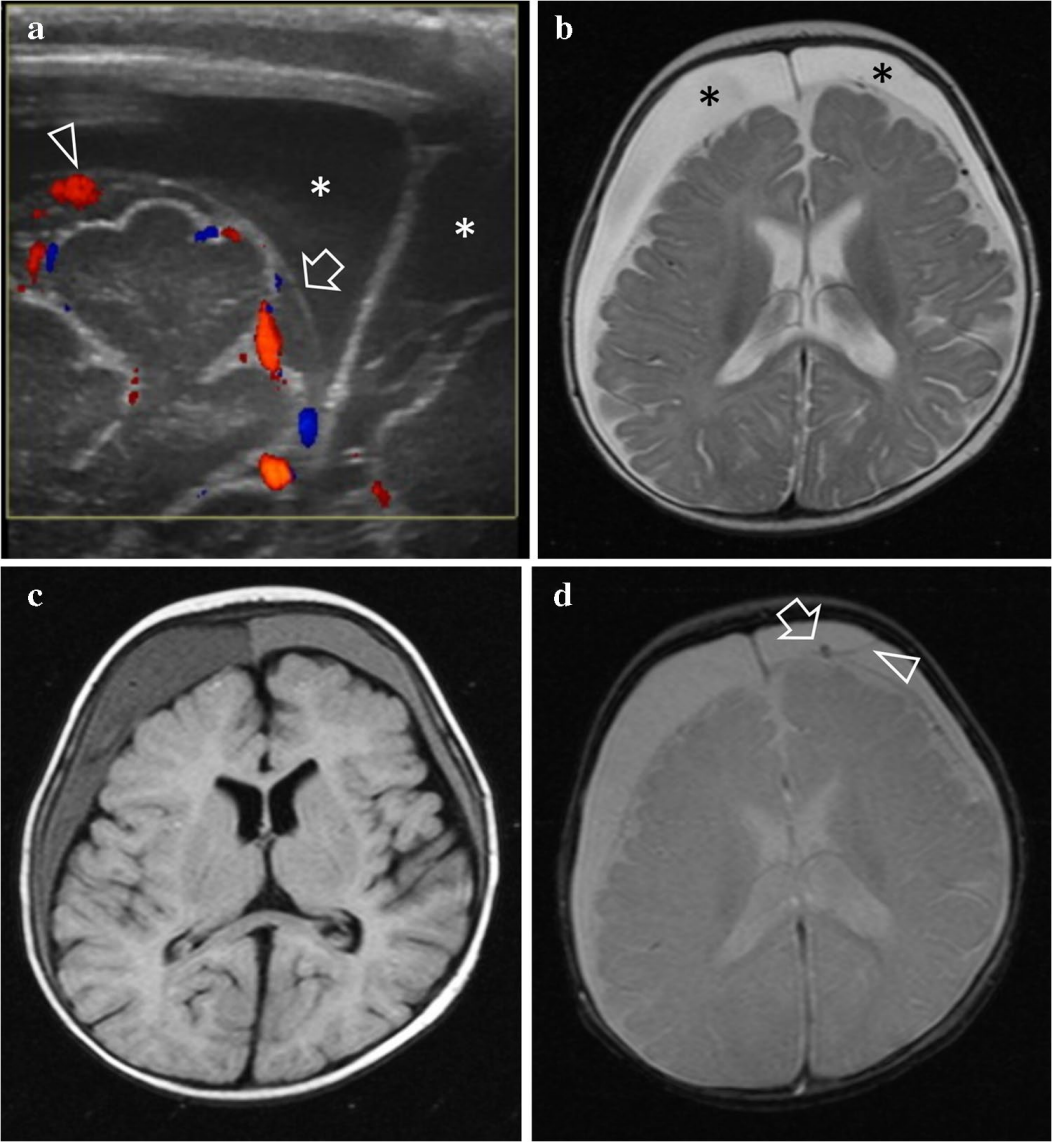
A 7-month-old ex-premature boy with progressive macrocephaly and normal development presenting with collections on ultrasound, confirmed by magnetic resonance imaging (MRI). Fundoscopy revealed retinal haemorrhages and skeletal survey revealed multiple fractures. The child was reported to authorities as physical abuse. **a** Coronal ultrasound with high-frequency linear transducer shows extra-axial anechoic collections (*) and vessels displaced against the brain surface (*arrowhead*) and below the arachnoid membrane (*arrow*). **b-d** MRI on the same day. **b** Axial T2-weighted sequence shows subdural hyperintense collections (*). **c** Axial fluid-attenuated inversion recovery image shows different intensity subdural collections. **d** T2* axial image shows a vessel-like structure (*arrow*) adjacent to a line (a*rrowhead*), thought to represent bridging vein thrombosis and a subdural membrane, respectively

On MR, this differentiation is easier because in BESS the brain is surrounded by one fluid compartment, exhibiting CSF signal intensity, containing free-traversing vessels (Fig. 5) [25]. In subdural collections/haematomas, at least two fluid compartments with different signal intensities may be seen and displaced vessels against the cortical surface are easier to identify (Fig. 6) [25, 27]. Radiologists should be aware of exceptional appearances of subdural collections in which bridging veins will remain visible with minimal displacement and subdural collections will show similar signal intensities to CSF. Utilization of T2* or susceptibility-weighted sequences for identification of blood products and routine performance of diffusion sequences for evaluation of brain cytotoxic oedema, maximize the diagnostic capabilities of this method in comparison to US and CT and should be an indispensable part of the MRI protocol in these children.

Radiologists should compare CSF density and signal intensity at the convexity to the respective density or signal intensity within the lateral ventricles to ensure they do not misinterpret bilateral symmetric hypodense subdural collections for BESS [26, 28].

## Subdural collections and their significance in the setting of BESS

Subdural haemorrhagic collections in children younger than 2 years, without any medical cause or a relevant history of trauma, should always raise concern for abusive head injury and should be extensively evaluated as per international guidelines [29–37]. Subdural collections can occur in children with BESS either spontaneously or because of accidental trauma [15, 16, 27, 38–40]. The theory of BESS predisposing to isolated subdural haemorrhage implicates over-stretching of the extra-axial blood vessels following brain translocation and has been supported by a mathematical model of the cranial vault [41]. The association between a greater depth of the subarachnoid space and the increased prevalence of such collections is controversial [15, 42]. Although enlarged subarachnoid spaces can be associated with subdural collections in children with macrocrania [39], long-term observations of infants with BESS as well as a finite element study indicated no increased risk for developing subdural haematomas [11, 43].

A review of 14 relevant studies describing imaging findings in children with BESS showed that subdural collection(s) occurred in 128 out of 1,705 children with BESS (7.5%) with a varying prevalence ranging from 0 to 42.1% (Table 1) [8, 12, 15, 16, 27, 39, 42, 44–50]. A review of 16 relevant studies mentioning co-existence of subdural collections with BESS, showed that only 83 out of 191 children with BESS and subdural collections were further evaluated with skeletal surveys and/or fundoscopy and 28/83 (33.7%) also had concomitant injuries, including extensive retinal haemorrhages and/or fractures (Table 1) [8, 12, 15, 16, 27, 38, 39, 42, 44–52]. In Table 1, the different prevalence of subdural collections among infants with BESS and of concomitant suspicious injuries among different studies can be attributed to the diversity of imaging modalities with different sensitivities in the detection of subdural collections and the diversity of practices among institutions in the investigation of such children. It is important to understand that in some of these historically important publications in Table 1, images produced with CT and MRI scanners of previous generations, it might have been difficult to determine whether the enlarged extra-axial spaces were actually subarachnoid or potentially subdural. This might also factor into the prevalence range variation stated. It should also be emphasized that a visible subdural space in the context of BESS, mentioned in the recent literature as a common finding of approximately 1 mm width [50] is not a synonym for a subdural haematoma discussed herein.

**Table 1:** Description of studies providing the prevalence of subdural collections in children with benign enlargement of the subarachnoid space (BESS) (*n* =14) and summarising the presence and results of investigations for concomitant injuries/abuse in children with subdural hematomas/collections and features of BESS (*n* =17)

Authors	Number of patients with BESS (*N*=1705)	Patients with subdural effusions	Patients with hemorrhagic subdural collections	Patients with subdural collections (effusions or haemorhhagic subdurals) *N*=133. Prevalence of subdurals in BESS 128/1705 (7.3%)	Concomitant injuries	Modalities for identification of SD in the setting of BESS	Investigations in children with SD and BESS for possible abuse	Number of children with SD referred to MDT meeting	Age of children with SD and BESS	Children with SD, BESS and concomitant injuries [suspicious RH and/or fractures]
Mori et al. 1992 [44]	20	0	3	3 (15%)	n/a	CT and MRI	Not mentioned	Not mentioned	2 to 30 months in age, with a mean age of 8.6 months	Unknown
Wilms et al. 1993 [27]	19	5	3	8 (42.1%)	One child with recent trauma (fall from dressing table), 3 difficult deliveries, 3 symptomatic	Contrast-enhanced CT, MRI	Not mentioned-not investigated	Not mentioned	7. 7 ± 4.1 months	Unknown
Alper et al. 1999 [45]	13	0	0	0 (0%)	n/a	MRI	n/a	n/a	n/a	n/a
Hansen et al. 1999 [51]	n/a	n/a	n/a	34 (n/a)	17 children had concomitant injuries	CT and MRI	Skeletal survey, fundoscopy, clinical investigations in 34 children	all	<2 years	*N*=17 (50%)
Laubscher et al. 1990 [46]	22	3	0	3 (13.6%)	“We are fairly confident that our three described infants with spontaneous subdural collection were not battered children”	CT, air pneumoencephalography, US	Not mentioned	Not mentioned	18 weeks-5 months	Unknown
Ravid & Maytal. 2003 [52]	n/a	0	3	3 (n/a)	none	CT and MRI	Skull surveys and fundoscopy in 3 children	Not mentioned	3,5,7 months	*N*=0
Mcneely et al. 2006 [38]	n/a	0	7	7 (n/a)	Abuse cases were excluded. 2 cases with accidental trauma	CT and MRI	extensive investigation and interview process done in 4 children, also fundoscopy	7 (100%)	3.6 -17.8 months of age	Unknown (abuse cases with BESS and SD collections excluded)
Yew et al. 2011 [8]	99	0	4	4 (4%)	negative screens for abusive head injury (implied in all 4 patients)	Not mentioned	Skeletal surveys and fundoscopy in 4 children	n/a	1–16 months (median 6.5 months)	*N*=0 (0%)
Ghosh & Ghosh. 2011 [47]	45	0	9	9 (20%)	1 child with fractures	CT and MRI	Skeletal survey (n=8), fundoscopy (n=6) social worker interview	1 patient with multilevel fractures. Fundoscopy negative when performed	Study included children <3 years. All patients with BESS and SD were 3 months-2 years old	*N*=1 (11%)
McKeag et al. 2013 [16]	177	0	4	4 (2.3%)	One patient with 2 healing rib fractures, distal radius fracture. Fundoscopy normal. Re-review of abdominal radiographs obtained for vomiting and altered mental status 4.5 months ago identified multiple healing rib fractures	MRI or CT. 33 children with BESS diagnosed via ultrasonography alone were excluded-none had SD collections	Brain MRI or CT, skeletal survey, fundoscopy in 4 children	4 children, 1 reported to a state agency	<2 years	*N*=1 (25%)
Greiner et al. 2013 [39]	108	4	2	6 (5.6%)	1 child had concerning retinal haemorrhages, 2 reported for abuse	CT and MRI	2 had initial and follow-up skeletal survey. 4 had fundoscopy	2 referred and reported to a state agency	<2 years	*N*=1 (18%)
Marino MA et al. 2014 [12]	5	0	1	1(20%)	n/a	CT and MRI	Not mentioned	Not mentioned	Average age 16 months	Unknown
Tucker et al. 2016 [15]	311	18	0	18 (5.8%)	One child with haemorrhagic subdural collection investigated for abuse-no concomitant injuries found	US, CT, limited single-shot fast spin echo (T2-weighted) study, complete MRI	Brain MRI or CT, skeletal survey, fundoscopy in 1 child	3 children examined by child abuse specialist, 1 reported to a state agency	<2 years	Unknown
Haws et al. 2017 [48]	84	0	2	2 (2.4%)	n/a	US, CT, MRI	Not mentioned	Not mentioned	mean age at diagnosis: 6.5 months	Unknown
Lee et al. 2017 [49]	213	4	16	20 (9.4%)	No discrepancy between the presence of subdural haemorrhage and the offered history of trauma, no concomitant injuries, no parental delay in seeking medical attention, no inappropriate/inconsistent reaction of parents, no history of a dysfunctional family or a suspicion of child abuse	CT (N=7 patients), MRI (N=12 patients), or brain US (N=1 patient)	Fundoscopy n=4. Skeletal surveys not mentioned	“If radiologic evidence of (craniocerebral?) trauma or a clue of child abuse was absent, we did not perform routine eye examination” Multidisciplinary team not mentioned	1–16 months (median 6.5 months	Unknown
Alshareef et al. 2022 [50]	109	0	11	11 (10%)	1 child was considered abuse, additional injuries not described	Fast brain MRI, regular MRI	Skeletal surveys and fundoscopy in 10 children	Not mentioned	8 + 4.6 months	*N*=1 (9%)
Holste KG, et al. 2022 [42]	480	0	58	58 (12.1%)	Fractures, retinal hemorrhages, positive diagnosis based on social work and child protection team investigations	MRI	Skeletal survey, fundoscopy, investigation by child protection team in 36 children	36 children referred to child protection team	0.8–24 months	*N*=7 (12%)

*MDT* multidisciplinary meeting, *n/a* not applicable, *N* number of patients, *n* number of studies, *RH* retinal haemorrhages, *SD* subdural collections

In infants with macrocephaly and subdural collections, the possibility of abusive injury may be supported by the presence of concomitant suspicious injuries in various organs and sites: co-existing parenchymal injuries or cytotoxic oedema, bridging vein thrombosis/rupture, subdural collections in different locations (around right frontal lobe, around left frontal lobe, convexity, interhemispheric fissure, posterior fossa), spinal injuries (including ligamentous injuries and subdural spinal haematomas), unexplained fractures, especially classic metaphyseal lesions (CMLs), rib and skull fractures [23, 29–32, 53–62]. Skin, oral and genital stigmata are extremely important to identify, strongly supporting the hypothesis of abuse in the absence of any additional finding in a child with BESS and subdural collection(s) [63–65]. A relevant social history disclosing factors and conditions that might place a child at risk for maltreatment and a previous history of unexplained or frequent trauma to the same patient or household members, a delay in seeking help and a changing history are also red flags for physical abuse [10, 64]. Apnoea, loss of consciousness, and death, to our knowledge, have not been described in infants with BESS, as opposed to infants with abusive head trauma [10, 35, 53]. Indeed, the hypothesis of subdural collection in the setting of BESS does not provide explanations for the presence of the concomitant injuries described above [51].

On the other hand, the presence of a homogeneous subdural collection, without clots over the vertex in particular, with continuous bridging veins in a macrocephalic asymptomatic infant with large subarachnoid spaces, with the expected head growth curve and lack of the aforementioned concomitant suspicious injuries on craniocerebral structures, bones (including vertebrae), skin/soft tissues, fundus, etc., favours the hypothesis of BESS associated with incidental subdural collections [15, 38].

## Topics requiring further studies

Non-specific signs including occasional seizures of less than 5 min duration, twitching, fussiness, crying, bulging fontanelle, and drowsiness cannot, when they are isolated, be used as discriminators between abusive head trauma and BESS with spontaneous subdural collections because they have been reported in both [9, 35, 51, 66]. It is not clearly known at present whether these signs are incidentally seen in BESS i.e., during a benign infectious intercurrent process or other neurological disease or if they can occur per se in BESS. Importantly, not only symptomatic but even asymptomatic or minimally symptomatic children with subdural collections and BESS may have concomitant injuries suspicious of abuse in up to 35% of cases when systematically screened according to the recommended protocols for suspected abuse [51]. Further studies are required to specify the severity of clinical signs and symptoms in children with subdural haemorrhages and BESS in an attempt to identify clinical discriminators and their potential association with fractures on skeletal surveys.

Macrocephaly with a fast-growing head, crossing percentiles, with a gradual or “abrupt” onset has been described in BESS [7, 17]. An abrupt onset of macrocephaly may also occur following acute abusive head trauma. The criteria for “abrupt onset” have not been yet specified in the literature. Traumatic large and bilateral subdurals may cause macrocrania and secondary enlargement of the subarachnoid spaces due to disruption of the arachnoid-dura interface, resulting in reduced CSF absorption by blocked arachnoid granulations [10, 24]. Further studies measuring head circumference with accuracy and comparing percentiles to international charts [3], may determine discriminating features of head circumference growth between the two entities, also keeping in mind that abusive head trauma may be repetitive [58].

Retinal haemorrhages have been described in approximately 85% of children with abusive head trauma and exceptionally in children with BESS [35, 67, 68]. Further studies are required to identify and clarify differences in occurrence and in imaging /fundoscopic patterns of retinal haemorrhages between the two populations.

Additional studies are required to define differences in characteristics of subdural collections in abused children with BESS compared to the subdural collections occurring in the setting of BESS following minor trauma, with regard to subdural collection depth, density on CT, or intensity on various MRI sequences and consequently stratify the risk of concomitant injuries. It is likely that the presence or absence of clots at the vertex related to the rupture of bridging veins is an important marker of severe trauma [35].

## Children with BESS and subdural collections: Who should we evaluate for abuse and how?

Evidence-based guidelines regarding children with subdural collections and BESS are currently lacking. Due to perceived social risks associated with abuse evaluation and perceived risks of radiation exposure from skeletal surveys, some physicians may choose not to conduct an abuse evaluation in children with BESS, thin subdural collections, and no or minimal neurological symptoms, assuming that these infants exhibit evidence of subdural collections in the setting of BESS and cannot also be victims of abusive head trauma [16, 51]. However, it is well-known that a significant proportion of children who suffered from severe abusive head trauma have been seen previously with findings which should have required further child abuse evaluation [69, 70]. Therefore, the “better safe than sorry” approach would dictate careful multi-disciplinary team assessment, including a thorough evaluation for skin (top-to-toe examination), oral, and genital abnormalities, investigation for previous history of unexplained trauma or frequent trauma to the same patient or household members, investigation of the family environment for factors and conditions that might place the child at risk for maltreatment (social work-up), laboratory tests for bleeding diathesis, fundoscopy and further imaging with a skeletal survey in all patients [10, 30, 51]. Importantly, fundoscopy and skeletal surveys should be performed as early as possible, since relative fundoscopic findings and CMLs may heal quickly, the latter within 2 weeks, the former within a few days, and as early as 3 days, depending on type and severity [40, 61, 68]. Some paediatricians may decide not to perform skeletal surveys if the remaining investigations are unremarkable. However, a skeletal survey may reveal key finding (s) even when fundoscopy is normal [53, 58] and is particularly indicated in symptomatic children, in the previous history of unexplained or frequent trauma in the same child or other members in the child’s household and in large, compressing, clearly haemorrhagic subdural collections [10, 16, 51]. The prevalence of skeletal injuries in children with prominent subarachnoid spaces and subdural collection(s) not examined with skeletal surveys and repeat skeletal surveys, is currently unknown.

**Fig. 7:**
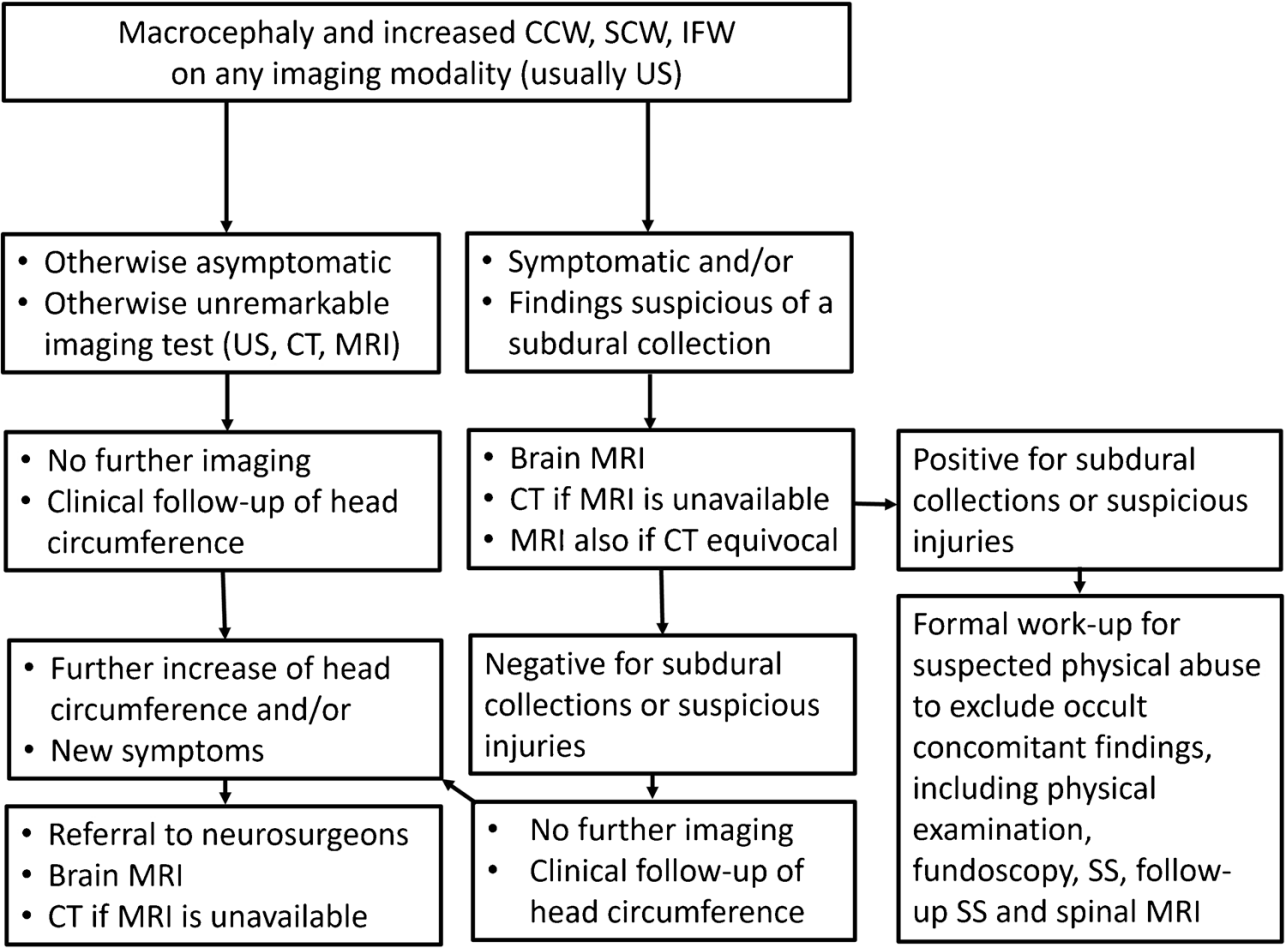
Clinical and imaging considerations in infants with macrocephaly and possible subdural collections. Children with benign enlargement of subarachnoid spaces (BESS) are more likely to be asymptomatic. Symptoms should alert physicians to the possibility of a subdural collection or another imaging finding not visible on ultrasound. *CCW* cranio-cortical width, *SCW* sino-cortical width, *IFW* interhemispheric fissure width, *US* ultrasound, *CT* computed tomography, *MRI* magnetic resonance imaging

In practice, the diagnosis of physical abuse cannot be medically ruled out without the exclusion of concomitant important findings in children with BESS and subdural collections [40]. Conversely, the diagnosis of physical abuse in an infant with BESS and an isolated subdural collection, especially when asymptomatic, is presumptive [69]. Not performing the full work-up may result in an unknown possibility of missing important occult findings. Consequently, based on our current understanding, we recommend to fully evaluate all these children as per national and international guidelines including initial and follow-up skeletal surveys (Fig. 7) [37]. Whole spine MRI, if included in the protocol of suspected physical abuse (Fig. 7), may reveal thoracolumbar injuries even when spinal radiographs are unremarkable [70].

Parents of children with BESS should be made aware of the possibility of subdural collection development and advised to take extra precautions to avoid minor trauma. This knowledge of the association of BESS and subdural collections may also alleviate unnecessary emotional trauma to parents/caregivers during interrogations to rule out abuse.

## Conclusions

Subdural collections in the setting of BESS are uncommon and large-scale studies are few.

1. Infants with subdural collection(s) and BESS: The diagnosis of abuse cannot be substantiated nor safely excluded without investigation for concomitant traumatic findings. The exact prevalence of occult injuries and abuse in these infants is unknown.
2. Infants with subdural haemorrhages (with or without BESS): Infants should be thoroughly evaluated, including initial and follow-up skeletal surveys even when fundoscopy, social work consult, and detailed clinical evaluation are unremarkable.

Subdural haematomas in children with macrocephaly and concomitant injuries cannot be attributed to the hypothesis of BESS, particularly if there is rupture of bridging veins at the vertex. The absence of concomitant and previous injuries and a generally benign clinical course supports the hypothesis of BESS-related collections rather than abuse. As a precaution, the children with BESS and subdural collections should be clinically followed for a few months by available specialists, ideally by the child abuse paediatrician and the child protection services, even if the diagnosis of abuse is not retained.

## Data Availability

All relevant information is already provided within the manuscript.

## References

[1] Wiig US, Zahl SM, Egge A (2017). Epidemiology of benign external hydrocephalus in Norway-a population-based study. Pediatr Neurol.

[2] Khosroshahi N, Nikkhah A (2018). Benign enlargement of subarachnoid space in infancy: a review with emphasis on diagnostic work-up. Iran J Child Neurol.

[3] Head circumference velocity https://www.who.int/tools/child-growth-standards/standards/head-circumference-velocity. Accessed 21 Nov 2022

[4] Alvarez LA, Maytal J, Shinnar S (1986). Idiopathic external hydrocephalus: natural history and relationship to benign familial macrocephaly. Pediatrics.

[5] Castro-Gago M, Pérez-Gómez C, Novo-Rodríguez MI (2005). Hidrocefalia externa idiopática benigna (efusión subdural benigna) en 39 niños: evolución natural y relación con la macrocefalia familiar [Benign idiopathic external hydrocephalus (benign subdural collection) in 39 children: its natural history and relation to familial macrocephaly]. Rev Neurol.

[6] Wilms G, Vanderschueren G, Demaerel PH (1993). CT and MR in infants with pericerebral collections and macrocephaly: benign enlargement of the subarachnoid spaces versus subdural collections. AJNR Am J Neuroradiol.

[7] Sainz LV, Schuhmann MU (2022). Subarachnomegaly-venous congestion of infancy. Childs Nerv Syst.

[8] Yew AY, Maher CO, Muraszko KM, Garton HJL (2011). Long-term health status in benign external hydrocephalus. Pediatr Neurosurg.

[9] Hellbusch LC (2007). Benign extracerebral fluid collections in infancy: clinical presentation and long-term follow-up. J Neurosurg.

[10] Caré MM (2021). Macrocephaly and subdural collections. Pediatr Radiol.

[11] Wittschieber D, Karger B, Niederstadt T et al (2015) Subdural hygromas in abusive head trauma: pathogenesis, diagnosis, and forensic implications AJNR 36:432–439. 10.3174/ajnr.A3989 PMC8013070 24948499

[12] Marino MA, Morabito R, Vinci S (2014). Benign external hydrocephalus in infants. A single centre experience and literature review. Neuroradiol J.

[13] Cinalli G, di Martino G, Russo C (2021). Dural venous sinus anatomy in children with external hydrocephalus: analysis of a series of 97 patients. Childs Nerv Syst.

[14] Proulx ST (2021). Cerebrospinal fluid outflow: a review of the historical and contemporary evidence for arachnoid villi, perineural routes, and dural lymphatics. Cell Mol Life Sci.

[15] Tucker J, Choudhary AK, Piatt J (2016). Macrocephaly in infancy: benign enlargement of the subarachnoid spaces and subdural collections. J Neurosurg Pediatr.

[16] McKeag H, Christian CW, Rubin D (2013). Subdural hemorrhage in pediatric patients with enlargement of the subarachnoid spaces. J Neurosurg Pediatr.

[17] Zahl SM, Egge A, Helseth E, Wester K (2011). Benign external hydrocephalus: a review, with emphasis on management. Neurosurg Rev.

[18] Libicher M, Tröger J (1992). US measurement of the subarachnoid space in infants: normal values. Radiology.

[19] Lam WW, Ai VH, Wong V, Leong LL (2001). Ultrasonographic measurement of subarachnoid space in normal infants and children. Pediatr Neurol.

[20] Prassopoulos P, Cavouras D, Golfinopoulos S, Nezi M (1995). The size of the intra- and extraventricular cerebrospinal fluid compartments in children with idiopathic benign widening of the frontal subarachnoid space. Neuroradiology.

[21] Wittschieber D, Karger B, Pfeiffer H, Hahnemann ML (2019). Understanding subdural collections in pediatric abusive head trauma. Am J Neuroradiol.

[22] Veyrac C, Couture A, Baud C (1990). Pericerebral fluid collections and ultrasound. Pediatr Radiol.

[23] Orrù E, Calloni SF, Tekes A (2018). The child with macrocephaly: differential diagnosis and neuroimaging findings. AJR.

[24] Zouros A, Bhargava R, Hoskinson M, Aronyk KE (2004) Further characterization of traumatic subdural collections of infancy. Report of five cases. J Neurosurg 100:512–518 10.3171/ped.2004.100.5.0512 15287465

[25] Wittschieber D, Karger B, Niederstadt T (2015). Subdural hygromas in abusive head trauma: pathogenesis, diagnosis, and forensic implications. AJNR.

[26] Knipe H, Toumpanakis D, Hacking C et al CT head (subdural window). Reference article, Radiopaedia.org (Accessed on 21 Nov 2022) 10.53347/rID-48361

[27] Wilms G, Vanderschueren G, Demaerel PH (1993). CT and MR in infants with pericerebral collections and macrocephaly: benign enlargement of the subarachnoid spaces versus subdural collections. AJNR.

[28] Meybodi KT, Habibi Z, Nejat F (2020). Temporary exacerbation of benign external hydrocephalus following minor head trauma. Childs Nerv Syst.

[29] Paine CW, Scribano PV, Localio R, Wood JN (2016). Development of guidelines for skeletal survey in young children with intracranial hemorrhage. Pediatrics.

[30] Kemp AM, Jaspan T, Griffiths J (2011). Neuroimaging: what neuroradiological features distinguish abusive from non-abusive head trauma? A systematic review. Arch Dis Child.

[31] Kemp AM (2002). Investigating subdural haemorrhage in infants. Arch Dis Child.

[32] Maguire SA, Kemp AM, Lumb RC (2011). Estimating the probability of abusive head trauma: a pooled analysis. Pediatrics.

[33] Greeley CS (2015). Abusive head trauma: a review of the evidence base. AJR.

[34] Wootton-Gorges SL, Soares BP, Alazrakin AL (2017). ACR appropriateness criteria ® suspected physical abuse—child. J Am Coll Radiol.

[35] Choudhary AK, Servaes S, Slovis TL (2018). Consensus statement on abusive head trauma in infants and young children. Ped Rad.

[36] Kelly P, John S, Vincent AL (2015). Abusive head trauma and accidental head injury: a 20-year comparative study of referrals to a hospital child protection team. Arch Dis Child.

[37] The Royal College of Radiologists, The Royal College of Paediatrics and Child Health, The Society and College of Radiographers (2018) The radiological investigation of suspected physical abuse in children. London. https://www.rcr.ac.uk/system/files/publication/field_publication_files/bfcr174_suspected_physical_abuse.pdf. Accessed 8 Feb 2023

[38] McNeely PD, Atkinson JD, Saigal G (2006). Subdural hematomas in infants with benign enlargement of the subarachnoid spaces are not pathognomonic for child abuse. AJNR.

[39] Greiner MV, Richards TJ, Care MM, Leach JL (2013). Prevalence of subdural collections in children with macrocrania. AJNR.

[40] Vinchon M, Delestret I, Defoort-Dhellemmes S (2010). Subdural hematoma in infants: can it occur spontaneously? Data from a prospective series and critical review of the literature. Childs Nerv Syst.

[41] Papasian NC, Frim DM (2000). A theoretical model of benign external hydrocephalus that predicts a predisposition towards extra-axial hemorrhage after minor head trauma. Pediatr Neurosurg.

[42] Holste KG, Wieland CM, Ibrahim M (2022). Subdural hematoma prevalence and long-term developmental outcomes in patients with benign expansion of the subarachnoid spaces. J Neurosurg Pediatr.

[43] Raul JS, Roth S, Ludes B, Willinger R (2008). Influence of the benign enlargement of the subarachnoid space on the bridging veins strain during a shaking event: a finite element study. Int J Legal Med.

[44] Mori K, Sakamoto T, Nishimura K, Fujiwara K (1993). Subarachnoid fluid collection in infants complicated by subdural hematoma. Child Nerv System.

[45] Alper G, Ekinci G, Yilmaz Y (1999). Magnetic resonance imaging characteristics of benign macrocephaly in children. J Child Neurol.

[46] Laubscher B, Deonna T, Uske A, van Melle G (1990). Primitive megalencephaly in children: natural history, medium term prognosis with special reference to external hydrocephalus. Eur J Pediatr.

[47] Ghosh PS, Ghosh D (2011). Subdural hematoma in infants without accidental or nonaccidental injury: benign external hydrocephalus, a risk factor. Clin Pediatr (Phila).

[48] Haws ME, Linscott L, Thomas C (2017). A retrospective analysis of the utility of head computed tomography and/or magnetic resonance imaging in the management of benign macrocrania. J Pediatr.

[49] Lee HC, Chong S, Lee JY (2018). Benign extracerebral fluid collection complicated by subdural hematoma and fluid collection: clinical characteristics and management. Childs Nerv Syst.

[50] Alshareef M, Tyler M, Litts C (2022). Prevalence of visible subdural spaces in benign enlargement of subarachnoid spaces in infancy: a retrospective analysis utilizing magnetic resonance imaging. World Neurosurg.

[51] Hansen JB, Frazier T, Moffatt M (2018). Evaluations for abuse in young children with subdural hemorrhages: findings based on symptom severity and benign enlargement of the subarachnoid spaces. J Neurosurg Pediatr.

[52] Ravid S, Maytal J (2003) External hydrocephalus: a probable cause for subdural hematoma in infancy. Pediatr Neurol 28:139-41 10.1016/s0887-8994(02)00500-3 12699866

[53] Piteau SJ, Ward MG, Barrowman NJ, Plint AC (2012). Clinical and radiographic characteristics associated with abusive and nonabusive head trauma: a systematic review. Pediatrics.

[54] Caré MM (2022). Parenchymal insults in abuse-a potential key to diagnosis. Diagnostics (Basel).

[55] Hahnemann ML, Kinner S, Schweiger B (2015). Imaging of bridging vein thrombosis in infants with abusive head trauma: the “Tadpole Sign”. Eur Radiol.

[56] Adamsbaum C, Rambaud C (2012). Abusive head trauma: don't overlook bridging vein thrombosis. Pediatr Radiol.

[57] Zuccoli G, Khan AS, Panigrahy A (2017). In vivo demonstration of traumatic rupture of the bridging veins in abusive head trauma. Pediatr Neurol.

[58] Adamsbaum C, Grabar S, Mejean N, Rey-Salmon C (2010). Abusive head trauma: judicial admissions highlight violent and repetitive shaking. Pediatrics.

[59] Choudhary AK, Ishak R, Zacharia TT, Dias MS (2014). Imaging of spinal injury in abusive head trauma: a retrospective study. Pediatr Radiol.

[60] Orman G, Kralik SF, Desai NK (2022). An in-depth analysis of brain and spine neuroimaging in children with abusive head trauma: beyond the classic imaging findings. AJNR.

[61] Marine MB, Forbes-Amrhein MM (2021). Fractures of child abuse. Pediatr Radiol.

[62] Offiah A, van Rijn RR, Perez-Rossello JM, Kleinman PK (2009). Skeletal imaging of child abuse (non-accidental injury). Pediatr Radiol.

[63] Hobbs CJ, Bilo RAC (2009). Nonaccidental trauma: clinical aspects and epidemiology of child abuse. Pediatr Radiol.

[64] Stray-Pedersen A, Vollmer-Sandholm MJ, Aukland SM (2022). Re-evaluation of abusive head trauma in Norway appears flawed. Acta Paediatr.

[65] Ludwig S, Warman M (1984). Shaken baby syndrome: a review of 20 cases. Ann Emerg Med.

[66] Togioka BM, Arnold MA, Bathurst MA (2009). Retinal hemorrhages and shaken baby syndrome: an evidence-based review. J Emerg Med.

[67] Piatt JH (1999). A pitfall in the diagnosis of child abuse: external hydrocephalus, subdural hematoma, and retinal hemorrhages. Neurosurg Focus.

[68] Pierre-Kahn V, Roche O, Dureau P (2003). Ophthalmologic findings in suspected child abuse victims with subdural hematomas. Ophthalmology.

[69] Pittman T (2003). Significance of a subdural hematoma in a child with external hydrocephalus. Pediatr Neurosurg.

[70] Karmazyn B, Reher TA, Supakul N (2022). Whole spine MRI in children with suspected abusive head trauma. AJR.

